# Editorial: Molecular pathology and computational image analyses in gynecologic malignancies

**DOI:** 10.3389/fonc.2022.1082220

**Published:** 2022-11-21

**Authors:** Umberto Malapelle, Sandra Orsulic

**Affiliations:** ^1^ Department of Public Health, University of Naples Federico II, Naples, Italy; ^2^ United State (US) Department of Veterans Affairs, Greater Los Angeles Healthcare System, Los Angeles, CA, United States; ^3^ Department of Obstetrics and Gynecology, David Geffen School of Medicine, University of California Los Angeles, Los Angeles, CA, United States; ^4^ Jonsson Comprehensive Cancer Center, University of California Los Angeles, Los Angeles, CA, United States

**Keywords:** cervical cancer, ovarian cancer, endometrial cancer, computational pathology, molecular pathology, imaging, gynecologic, artificial intelligence

Gynecological malignancies represent an important public health problem due to high cancer-related mortality. Despite improvements in diagnosis and treatment, gynecological malignancies account for about 40% of all cancer incidence and more than 30% of all cancer-related mortality in women worldwide (1, 2). Recent advances in molecular pathology, digital pathology, and computational imaging are paving way for new approaches to the diagnosis and prediction of clinical outcomes in gynecological malignancies (Orsulic et al.; 3–7). The continuously increasing capacity to store and analyze digital data enables applications of new technologies, such as artificial intelligence- and deep learning-assisted image analyses to generate thousands of image features and other non-biased continuous quantifiable variables that can be readily integrated with other -omic platforms (8). Future clinical application of such features combined with improvements in molecular pathology may be an arrow in the quiver of pathologists in diagnosis and guiding patient treatment and management decisions.

In this Research Topic of Frontiers in Oncology, we attempt to address some major advances in molecular and computational pathology in gynecological malignancies.

Endometrial cancer is the most common gynecological cancer in developed countries. Ovarian preservation treatment (OPT) is an option for patients of child-bearing age and with early-stage endometrial cancer, however, the benefits need to be carefully assessed against the risk of cancer progression. In order to assist radiologists in assessing the depth of myometrial invasion and selecting eligible patients for OPT, Yan et al. developed and validated a radiomics nomogram based on multi-parametric magnetic resonance imaging and the least absolute shrinkage and selection operator algorithm. Molecular analyses are a useful tool in the characterization and classification of recently identified malignancies, such as mesonephric-like adenocarcinoma (MLA), a rare and aggressive neoplasm that mostly arises in the uterine corpus. Ma et al. analyzed molecular alterations in four MLA cases and identified several immunohistochemical markers as well as recurrent mutations in PIK3CA, KRAS, and PTEN, which not only support the classification of these malignancies as Müllerian in origin with mesonephric differentiation but also provides important leads for targeted therapies.

Cervical cancer is the leading cause of gynecological tumor-related mortality worldwide and the second most common malignancy in women. Human papilloma virus (HPV) infection and integration within the human genome is the primary cause of cervical cancer. Integration can disrupt the function of nearby genes, including oncogenes and tumor suppressor genes, and cause genomic rearrangements and instability (9). However, the potential intra-tumoral heterogeneity in integration sites has not been explored. Using an optimized dual-color fluorescence *in situ* hybridization (FISH) method to detect HPV integration sites in formalin-fixed, paraffin-embedded cervical cancer samples, Xiong et al. showed that cervical cancer may comprise subpopulations of cells with distinct integration sites that are otherwise indistinguishable by cell and nuclear morphology. Beyond diagnosis, molecular testing may be adopted for prognostic and predictive purposes in cervical cancer patients. Ma et al. showed that the upregulation of *CD27*, *TNF*, *TNFRSF12A*, *TNFRSF13C*, and *TNFRSF9* and the downregulation of *EDA* mRNA expression levels may serve as prognostic biomarkers of cervical cancer because they are associated with the immunotherapy response of these patients. In addition to molecular tools, cervical cancer patients may benefit, in particular in the field of screening and early diagnosis, from artificial intelligence-based medical tools. As summarized by Hou et al., clinical application of artificial intelligence may reduce turn-around time and the need for professional and technical personnel as well as eliminate human bias in evaluating subjective variables.

Ovarian cancer is a complex gynecological malignancy with a high mortality rate. To shed light on the molecular heterogeneity of this disease, Chen et al. explored a novel molecular phenotyping method for ovarian cancer subtypes based on metabolic genes through a comprehensive analysis of genomic data. The authors identified three different molecular subtypes (C1, C2, and C3) of ovarian carcinomas, which improved our understanding of the molecular characteristics of human ovarian cancer and uncovered new potential therapeutic targets. Wang et al. reviewed the role of the glyoxalase system as a marker for diagnosis and a novel target for antitumor therapy in breast, ovarian, endometrial, and cervical cancers. Although research in the past two decades has revealed that the fallopian tube is the likely precursor tissue for most epithelial ovarian cancers, the cell-intrinsic and microenvironmental conditions that lead to epithelial cell transformation into serous tubal intraepithelial carcinoma (STIC) are unknown. Wu et al. used computational image analyses to identify potential morphometric and topologic differences in fallopian tubes with and without STIC lesions. They showed that STIC lesions were accompanied by global stromal alterations and age-associated reorganization of tubal secretory and ciliated cells, which may provide a favorable microenvironment for cancer initiation. Another poorly understood aspect of ovarian cancer is metastasis outside of the peritoneal cavity. Brain metastases are rare in ovarian cancer, possibly because patients succumb to the peritoneal disease before the cancer has a chance to metastasize to other parts of the body. Using a spatially-oriented single-cell proteomics platform, Pejovic et al. identified cell populations that are shared between primary low-grade serous ovarian carcinomas and brain metastases, suggesting that cells predetermined for brain metastasis may exist in the early stages of cancer development. They also identified several brain metastasis-specific oncogenic and immunosuppressive pathways that could be used for targeted therapy.

Overall, this Research Topic has highlighted some major advances in molecular and computational pathology in gynecological malignancies. Ongoing research is warranted to improve the clinical outcome of these patients.

## Author contributions

All listed authors have made a substantial, direct, and intellectual contribution to the work and approved it for publication.

## Funding

SO is supported by the Veterans Affairs Merit Award VA-ORD I01 BX004974, the Department of Defense Award W81XWH2210631, the Iris Cantor NCATS UCLA CTSI grant UL1TR00188, and the Sandy Rollman Ovarian Cancer Foundation. UM declared no specific grant for this review from any funding agency in the public, commercial, or not-for-profit sectors.

## Conflict of interest

UM has received personal fees as consultant and/or speaker bureau from Boehringer Ingelheim, Roche, MSD, Amgen, Thermo Fisher Scientifics, Eli Lilly, Diaceutics, GSK, Merck and AstraZeneca, unrelated to the current work.

The remaining author declares that the research was conducted in the absence of any commercial or financial relationships that could be construed as a potential conflict of interest.

## Publisher’s note

All claims expressed in this article are solely those of the authors and do not necessarily represent those of their affiliated organizations, or those of the publisher, the editors and the reviewers. Any product that may be evaluated in this article, or claim that may be made by its manufacturer, is not guaranteed or endorsed by the publisher.
